# The impact of neighborhood environment on physical activity among older adults: chain mediating roles of self-efficacy and outcome expectations

**DOI:** 10.3389/fnagi.2025.1730899

**Published:** 2025-12-08

**Authors:** Lei Ying, Qingqing Yang, Jizhe Yu, Xiaoyun Liao, Wenming Fan

**Affiliations:** 1 School of Wushu, Chengdu Sports University, Chengdu, China; 2 Chinese Wushu Academy, Beijing Sports University, Beijing, China

**Keywords:** physical activity, neighborhood environment, self-efficacy, outcome expectations, the chain mediation effect

## Abstract

**Background:**

With the acceleration of population aging and the increasingly prominent problem of insufficient physical activity among older adults, how to effectively promote physical activity participation among older adults has become an important issue in the field of public health. Neighborhood environment, as the primary setting for daily living among older adults, exerts considerable influence on their physical activity engagement; however, the underlying mechanisms remain inadequately understood. Grounded in Social Cognitive Theory, this study aimed to examine the impact of neighborhood environment on physical activity among older adults and to test the chain mediating effects of self-efficacy and outcome expectations.

**Methods:**

This study employed a cross-sectional design to collect data on physical activity participation from 937 older adults residing in 12 communities across Chengdu, Sichuan Province, China, through questionnaire surveys. Assessment instruments included the Neighborhood Environment Scale, International Physical Activity Questionnaire, Self-Efficacy Scale, and Physical Activity Outcome Expectations Scale to comprehensively evaluate participants’ environmental perceptions, physical activity levels, and psychological cognitive characteristics. Following data collection, statistical analyses were conducted using SPSS 26.0, with structural equation modeling (AMOS) and Bootstrap methods employed to test potential mediating effects and ensure the reliability of results.

**Results:**

The analyses revealed significant positive correlations among neighborhood environment, physical activity, self-efficacy, and outcome expectations. Specifically, neighborhood environment demonstrated a significant direct effect on physical activity among older adults, indicating that favorable neighborhood environments directly facilitate physical activity participation. Furthermore, self-efficacy and outcome expectations exhibited a chain mediating effect between neighborhood environment and physical activity, whereby neighborhood environment indirectly influenced physical activity levels through enhancing individual exercise self-efficacy and outcome expectations.

**Conclusion:**

This study elucidates the underlying mechanisms between neighborhood environment and physical activity among older adults, Neighborhood environment is not only directly associated with physical activity levels among older adults but also indirectly influences physical activity through two psychological cognitive mediators: self-efficacy and outcome expectations.

## 1 Introduction

Physical activity, defined as any bodily movement produced by skeletal muscle contraction that significantly increases energy expenditure, represents a critical factor in maintaining physical and mental health and preventing chronic diseases among older adults (Taylor et al., 2004). Adequate physical activity can effectively prevent and control cardiovascular and cerebrovascular diseases, improve physical fitness, enhance mental health, and consequently improve quality of life in older populations (Wong et al., 2023). However, with the acceleration of urbanization and lifestyle transitions, global physical activity levels among older adults have exhibited a declining trend. The World Health Organization reports that approximately 27.5% of older adults worldwide fail to meet the recommended physical activity levels (Guthold et al., 2018), rendering insufficient physical activity a significant public health concern closely associated with multiple chronic conditions including obesity (Hou et al., 2025), cardiovascular diseases (Ciumărnean et al., 2021), and diabetes (Huang and Lu, 2024). Consequently, in-depth investigation of the factors influencing physical activity among older adults and their underlying mechanisms has become particularly urgent.

Under the background of the Healthy China strategy, multiple Chinese policies have continuously focused on national health, with a series of documents clearly establishing the important position of physical activity and promoting comprehensive coverage of fitness facilities across county, township, and village levels. The Healthy China 2030 Planning Outline deeply integrates health concepts into urban development, with particular emphasis on the foundational role of neighborhood environment. Among the various factors influencing physical activity participation in older adults, neighborhood environment, as an essential setting for aging in place and daily living, exerts significant influence on physical activity levels (Chaudhury et al., 2016). Neighborhood environment encompasses both physical environmental elements, including the accessibility and availability of fitness facilities, convenience and safety of roads, and adequacy of open spaces, as well as social environmental factors such as opportunities for social participation and interaction (Pan et al., 2022). With societal development, expectations for neighborhood environment quality have progressively increased. Research indicates that favorable neighborhood environments are associated with physical activity levels among older adults (Saelens and Papadopoulos, 2008). Neighborhoods characterized by good walkability, comprehensive living service facilities, adequate open spaces, and pleasant environmental design can promote greater resident participation in outdoor activities and yield enhanced health benefits (Barnett et al., 2017). Neighborhood road conditions, sports facility provisions, social activity opportunities, and household air pollution can all influence physical activity levels among older adults (Chaudhury et al., 2012; Guo et al., 2024; Dong et al., 2025). Older adults residing in favorable neighborhood environments can more readily access physical activity opportunities and maintain active lifestyles (Portegijs et al., 2023). They are better equipped to cope with life stressors and promptly alleviate states of tension and depression (de Oliveira et al., 2019). Conversely, older adults in inadequate neighborhood environments may encounter barriers to physical activity, with potential adverse effects on cognitive function and physical and mental health (Krell-Roesch et al., 2021; Balfour and Kaplan, 2002).

Although previous studies have established a positive correlation between neighborhood environment and physical activity among older adults, the underlying mechanisms warrant further investigation. Merely confirming the direct relationship between these two factors cannot fully elucidate how neighborhood environment influences physical activity in older adults, nor can it provide precise intervention targets for practical applications. Based on the above literature review, it can be observed that existing research has predominantly focused on the direct association between community environment and physical activity among older adults, while the exploration of underlying mechanisms remains relatively insufficient. This research gap suggests the need to introduce an appropriate theoretical framework to systematically elucidate the internal processes and psychological pathways through which environmental factors influence behavior. Among the various relevant theories, social cognitive theory offers a critical theoretical framework for understanding how environmental factors influence behavior (Bandura, 2014). This theory posits that environmental factors do not directly determine behavior but rather operate through individual cognitive processes (Rodrigues et al., 2023). In the field of physical activity research, self-efficacy and outcome expectations are regarded as important cognitive mediators linking environmental factors with behavioral outcomes (Egele et al., 2025). Self-efficacy, a fundamental component of Social Cognitive Theory, refers to individuals’ confidence or beliefs in their capability to achieve goals in specific domains (Schunk and DiBenedetto, 2021). Self-efficacy represents an essential psychological factor influencing individual behavioral choices, effort expenditure, and persistence. In the domain of physical activity, self-efficacy is recognized as a crucial variable predicting individual physical activity participation (McAuley, 1993). Research demonstrates that self-efficacy exhibits a significant positive correlation with physical activity levels, with individuals possessing high self-efficacy demonstrating greater propensity to engage in physical activity and maintain long-term adherence (Xie et al., 2025). Self-efficacy not only directly influences individual physical activity behavior but also moderates the impact of external environmental factors on behavior (Oba et al., 2024). Individuals with high self-efficacy can better utilize neighborhood environment resources and overcome barriers to physical activity. Training and interventions can effectively enhance individual self-efficacy. Research indicates that self-efficacy serves as an important predictor of individual subjective well-being (Caprara and Steca, 2005). Individuals with high self-efficacy exhibit stronger adaptive capacity and more positive behavioral performance when confronting challenges. Therefore, favorable self-efficacy contributes to physical activity participation, adaptive behavior development, and positive cognitive attitudes among older adults (Toros et al., 2023). Outcome expectancy refers to individuals’ subjective anticipation and evaluation of potential outcomes following the execution of specific behaviors (Schunk and DiBenedetto, 2020). It constitutes an important construct within Social Cognitive Theory and plays a pivotal role in influencing individual behavioral decision-making processes. In physical activity research, outcome expectations are defined as individuals’ anticipation of potential physical, psychological, and social benefits following physical activity participation (Williams et al., 2005). Research demonstrates that outcome expectations exhibit a significant positive correlation with physical activity participation, with positive outcome expectations facilitating individual physical activity behavior (Bohlen et al., 2022). Outcome expectations represent a crucial predictor of physical activity behavior change. Individuals with positive outcome expectations demonstrate greater propensity to engage in physical activity and maintain persistence when confronting difficulties. Individuals with high outcome expectations manifest stronger motivation and more sustained behavioral patterns in physical activity participation (Wang et al., 2025).

**Figure 1:**
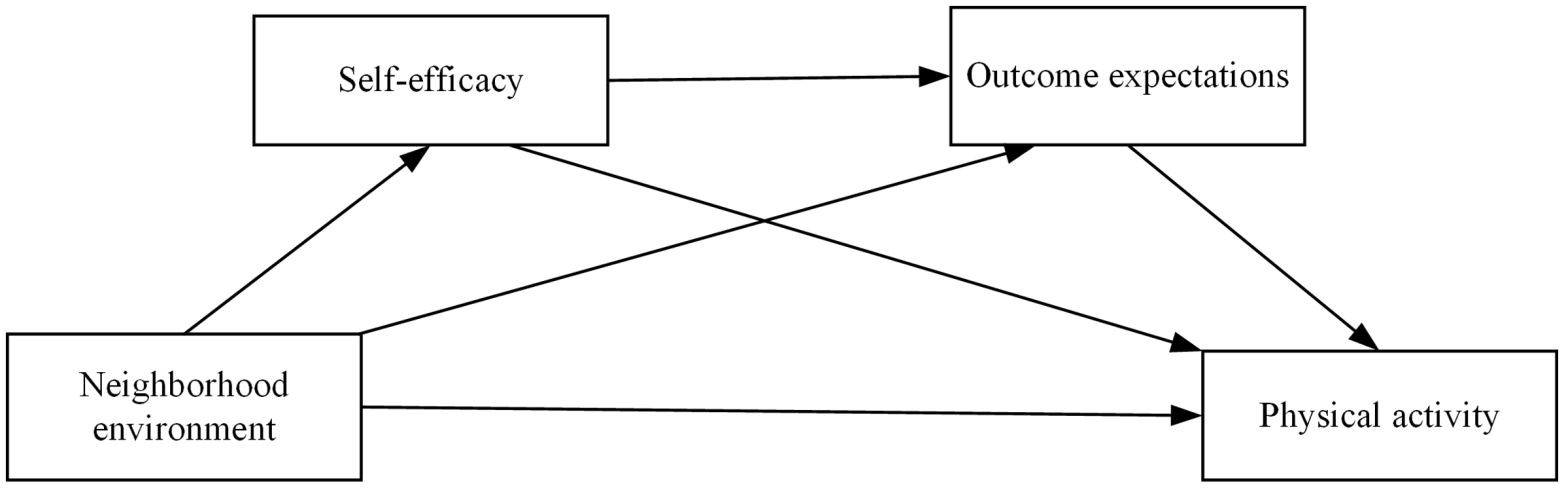
A hypothetical model of neighborhood environment affecting physical activity.

Notably, a sequential relationship may exist between self-efficacy and outcome expectations, whereby individuals with high self-efficacy tend to develop more positive outcome expectations (Woreta et al., 2025). However, the sequential mediating process through which these two variables interact in the influence of neighborhood environment on physical activity among older adults has not been adequately examined. The present study addresses this research gap by analyzing the temporal sequence of cognitive development, wherein individuals must first establish confidence in their own capabilities (self-efficacy), subsequently form positive anticipations regarding behavioral outcomes (outcome expectations), and ultimately translate these cognitions into actual behavioral participation. This sequential cognitive process suggests the existence of a chain mediation effect, whereby neighborhood environment may form a complete psychological transmission pathway by sequentially influencing self-efficacy and outcome expectations, ultimately promoting enhanced physical activity levels among older adults. Specifically, when older adults perceive high-quality neighborhood environments, their confidence in exercise capabilities is enhanced (elevated self-efficacy), which in turn strengthens anticipations of positive exercise outcomes (enhanced outcome expectations), ultimately facilitating physical activity participation. Based on these observations, we propose the following research hypotheses. Figure 1 presents the conceptual model of the hypothesized relationships:

Hypothesis I: Neighborhood environment is positively associated with physical activity among older adults.

Hypothesis II: Self-efficacy mediates the relationship between neighborhood environment and physical activity among older adults.

Hypothesis III: Outcome expectations mediate the relationship between neighborhood environment and physical activity among older adults.

Hypothesis IV: Self-efficacy and outcome expectations exert a chain mediating effect in the relationship between neighborhood environment and physical activity among older adults.

## 2 Materials and methods

### 2.1 Research design

To obtain the primary data required for this study, researchers designed a self-administered questionnaire based on existing literature and validated data collection instruments, focusing on assessing neighborhood environment, physical activity participation, self-efficacy, and outcome expectations among older adults. The research team conducted questionnaire surveys among older adults from 12 communities in Chengdu, China, from May 15, 2025, to August 15, 2025. The questionnaire comprised the Neighborhood Environment Scale, International Physical Activity Questionnaire, Self-Efficacy Scale, and Physical Activity Outcome Expectations Scale. All data collection was performed under standardized conditions to minimize data bias. Each questionnaire required approximately 15–25 min to complete, and all invited participants participated voluntarily. The Ethics Committee of Chengdu Sport University approved this project ([2025]193), and the research procedures adhered to the ethical requirements stipulated in the Declaration of Helsinki.

### 2.2 Inclusion and exclusion criteria

The inclusion criteria for this study were: (1) permanent residents currently living in the study communities with a residence duration ≥1 year; (2) older adults aged 60 years and above, possessing basic capability to engage in physical activities (including daily work, daily transportation, household chores, and exercise practices such as Tai Chi, Baduanjin, Yijinjing, as well as leisure and recreational activities); (3) adequate Chinese reading comprehension ability to independently complete the questionnaire; (4) voluntary participation with full understanding of the research purpose, procedures, and risks, and provision of signed informed consent.

The exclusion criteria were: (1) involuntary participation or withdrawal request during the survey process; (2) presence of severe cognitive impairment, mental illness, or undergoing psychological treatment that might compromise the authenticity and validity of questionnaire responses; (3) history of serious illness or medical restrictions within the past 6 months that completely preclude participation in any form of physical activity; (4) poor questionnaire response quality, including incorrect or missing responses to key items, evident regular response patterns, or abnormal completion time (below 50% of the median completion time or exceeding 3 standard deviations); (5) temporary residents or transient population lacking adequate understanding and experience of the neighborhood environment.

### 2.3 Measurement tool design and reliability testing

#### 2.3.1 Neighborhood environment scale

The This study employed the Neighborhood Environment Scale developed by Chen and Ning (2015). The questionnaire comprises six dimensions: (1) External environment: fresh air, absence of noise, and cleanliness; (2) Landscape greening: public green spaces meeting usage requirements, diverse plant species, proper plant maintenance, attractive landscape design, and availability of rest areas within green spaces; (3) Community planning: outdoor activities free from vehicular interference, rational road planning, reasonable vehicle parking, absence of significant water accumulation after heavy rain, and availability of fitness facilities within the community; (4) Surrounding supporting facilities: convenient transportation, shopping, medical services, and schooling access; (5) Neighborhood relationships: communication with neighbors, good relationships, mutual trust, and mutual assistance; (6) Community interaction: frequent community-organized activities, suitable activities organized by the community, and regular participation in community activities. Each item was rated on a five-point Likert scale, where 1 represents “strongly disagree” and 5 represents “strongly agree,” with higher scores indicating greater neighborhood environment support for physical activity. In this study, the scale demonstrated a Cronbach’s *α* coefficient of 0.876.

#### 2.3.2 International physical activity questionnaire

This study utilized the International Physical Activity Questionnaire (IPAQ) (Craig et al., 2003), which comprises six components: daily work, daily transportation, household chores, exercise and training, leisure and recreational activities, and sedentary time and sleep duration. The questionnaire has demonstrated satisfactory reliability and validity in Chinese populations aged 60 years and above and is suitable for assessing physical activity in this demographic (Wang, 2018). Physical activity intensity was quantified by converting it to metabolic equivalent task minutes per week (MET-min/week) according to the IPAQ scoring and data processing guidelines. Physical activity levels were classified as follows: low level (<600 MET-min/week), moderate level (600–2,999 MET-min/week), and high level (≥3,000 MET-min/week).

#### 2.3.3 Self-efficacy scale

This study adopted the General Self-Efficacy Scale (GSES) developed by German clinical psychologist Schwarzer and colleagues (Schwarzer, 1997). The GSES has been translated into multiple languages and is widely utilized across various countries. The present study employed the Chinese version of the General Self-Efficacy Scale translated and revised by Wang and colleagues (Wang et al., 2001). The scale comprises 10 items rated on a four-point scale, ranging from “completely disagree” to “completely agree,” scored from 1 to 4 points. Based on the obtained scores, self-efficacy was classified into three levels: low level (10–19 points), moderate level (20–30 points), and high level (31–40 points). The Cronbach’s *α* coefficient for this scale in the present study was 0.887.

#### 2.3.4 Outcome expectations for exercise scale

This study employed the Chinese version of the Outcome Expectations for Exercise Scale (OEE), originally developed by Resnick et al. (2000) and validated in Chinese by Lee et al. (2011). The scale comprises 9 items assessing individuals’ expectations regarding potential positive outcomes from physical activity participation, including improvements in physical health, enhancement of psychological states, and increased social interaction. Items were rated on a five-point Likert scale ranging from “strongly disagree” to “strongly agree,” scored from 1 to 5 points, with higher scores indicating greater expectations for positive outcomes from physical activity. The Cronbach’s α coefficient for this scale in the present study was 0.912.

#### 2.3.5 Control variables

To validate the effects of neighborhood environment on physical activity among older adults, this study selected the following control variables based on relevant research to maximize internal validity and minimize interference from potential confounding factors. First, gender and age have been established as critical variables requiring control in research. Previous studies have demonstrated that gender exhibits significant differences in physical activity participation patterns, activity preferences, and self-efficacy formation among older adults (Roh and Chang, 2025). Age possesses important discriminative significance within the older adult population. Young-old and oldest-old adults demonstrate significant differences in activity capacity, gait characteristics, and cardiopulmonary metabolic energy expenditure (Chung et al., 2023). Second, education level reflects cognitive resources and health literacy levels among older adults. Older adults with different educational backgrounds exhibit significant differences in health awareness, information acquisition capabilities, and outcome expectation formation (Kwon and Kwon, 2025). Older adults with higher education may possess clearer cognition of the health benefits of physical activity, have more explicit requirements for neighborhood environments, and demonstrate greater proficiency in utilizing community resources to promote their physical activity participation. Finally, physical health status among older adults was included as a direct indicator of health condition. Healthy older adults differ significantly from those with illnesses in terms of physical activity capacity, activity motivation, and neighborhood environment needs (Gust, 2025). Illness not only restricts older adults’ activity capacity but may also diminish their self-efficacy and outcome expectation levels. The selection of these control variables adheres to best practices in older adult physical activity research, ensuring accurate identification of the unique contribution of neighborhood environment to physical activity through the mediating pathways of self-efficacy and outcome expectations, thereby enhancing the internal validity of research conclusions and the reliability of causal inference.

#### 2.3.6 Statistical analysis

Following organization of valid questionnaire data, analyses were conducted using SPSS 26.0 software. Correlation analysis and linear regression analysis were employed to examine the relationships among neighborhood environment, physical activity, self-efficacy, and outcome expectations. The Amos 24.0 software package was utilized to validate the model and test the structural validity of the scales. Currently, the Bootstrap method represents a commonly employed approach for testing mediation effects. This method involves repeated sampling from the original sample and examines the significance of mediation effect coefficients through 95% confidence intervals. Therefore, the present study employed the Bootstrap method to test whether self-efficacy and outcome expectations mediate the relationship between neighborhood environment and physical activity, as well as whether self-efficacy and outcome expectations exert a chain mediation effect in the relationship between neighborhood environment and physical activity.

## 3 Results

### 3.1 Validity testing

As the scales employed in this study were adapted from previously developed questionnaires, validation of their reliability and validity was necessary. To further examine the convergent validity and reliability of the scales, Average Variance Extracted (AVE) and Construct Reliability (CR) were utilized as evaluation parameters. AVE is commonly employed to reflect the convergent validity of scales and directly indicates the proportion of variance explained by latent variables that originates from measurement error. Higher AVE values indicate that a greater percentage of variance in measurement variables is explained by latent variables, with correspondingly lower measurement error. CR reflects whether all items within each latent variable consistently explain that latent variable. As shown in Table 1, the AVE values for all factors exceeded 0.5, demonstrating satisfactory model convergence. The CR values for all factors exceeded 0.7, confirming that items within each scale consistently explained their respective latent variables, indicating adequate construct reliability. In summary, the questionnaire employed in this study demonstrated high reliability and validity.

**Table 1:** Validity and reliability test of the questionnaires.

Variable	CR	AVE
NE	0.868	0.525
PA	0.737	0.513
SE	0.910	0.503
OE	0.920	0.559

CR, composite reliability; AVE, average variance extracted; NE, neighborhood environment; PA, physical activity; SE, self-efficacy; OE, outcome expectations.

### 3.2 Common method variance testing

To minimize common method bias, coded anonymous assessment procedures were implemented during data collection to control for sources of common method bias at the procedural level. Additionally, Harman’s single-factor test was conducted using SPSS 26.0 to perform exploratory factor analysis on all measurement items. Results revealed seven factors with eigenvalues greater than 1, with the first factor accounting for 30.52% of the variance, below the critical threshold of 40%. These findings indicate that common method bias was not a serious concern in the present study.

### 3.3 Descriptive statistics and correlation analysis

This study conducted a questionnaire survey of 1,000 older adults from 12 communities in Chengdu, Sichuan Province, China, between May and August 2025, yielding 937 valid responses. As shown in Table 2, physical activity levels were distributed as follows: high level 414 participants (44.2%), moderate level 388 participants (41.4%), and low level 135 participants (14.4%). Participants demonstrated balanced gender distribution, with 434 males (46.3%) and 503 females (53.7%). Regarding age distribution, participants aged 60–64 years comprised the largest group 350 participants (37.4%), followed by 65–69 years 238 participants (25.4%), 70–74 years 191 participants (20.4%), 75–79 years 140 participants (14.9%), and 80 years and above 18 participants (1.9%). Educational background distribution included middle school 277 participants (29.6%), primary school or below 256 participants (27.3%), high school or technical secondary school 224 participants (23.9%), junior college or vocational college 133 participants (14.2%), and bachelor’s degree or above 47 participants (5.0%). Monthly income distribution revealed 2,001–4,000 yuan 316 participants (33.7%), ≤2,000 yuan 308 participants (32.9%), 4,001–6,000 yuan 198 participants (21.1%), 6,001–8,000 yuan 84 participants (9.0%), and >8,000 yuan 31 participants (3.3%). Self-reported health status included very good 167 participants (17.8%), relatively good 176 participants (18.8%), fair 272 participants (29.0%), relatively poor 180 participants (19.2%), and very poor 142 participants (15.2%).

**Table 2:** Participant demographics.

Demographic category	Frequency	Percent%
Physical activity level		
Low level	135	14.4
Medium level	388	41.4
High level	414	44.2
Gender		
Male	434	46.3
Female	503	53.7
Age		
60–64	350	37.4
65–69	238	25.4
70–74	191	20.4
75–79	140	14.9
80 years and above	18	1.9
Education level		
Primary school and below	256	27.3
Middle school	277	29.6
High school or secondary vocational school	224	23.9
Junior college (including higher vocational education) education	133	14.2
Bachelor’s degree and above	47	5
Monthly income		
Below 2000	308	32.9
2001–4,000	316	33.7
4,001–6,000	198	21.1
6,001–8,000	84	9
Above 8,000	31	3.3
Physical health level		
Excellent	167	17.8
Good	176	18.8
Average	272	29
Poor	180	19.2
Very poor	142	15.2

This study focused on examining the overall scores of each model indicator without considering the subdivided dimensions of each indicator, using mean scores of each variable for Pearson correlation analysis. Descriptive statistics and correlation analysis results for the four variables of neighborhood environment, self-efficacy, outcome expectations, and physical activity among older adults are presented in Table 3. To assess the normality of data distribution, the table includes means, standard deviations, skewness, and kurtosis values. According to the criteria proposed by Kline (2023), data can be considered to satisfy the normality assumption when the absolute values of skewness are less than 3 and the absolute values of kurtosis are less than 10. As demonstrated in Table 3, considering both skewness and kurtosis indicators, all major variables in this study satisfy the normality assumption, supporting the utilization of subsequent parametric statistical analyses, including Pearson correlation analysis and structural equation modeling.

**Table 3:** Descriptive statistics and correlations for primary variables.

Variable	*M*	S.D.	Skew	Kurt	NE	SE	OE	PA
NE	3.478	0.67	−0.757	0.433	1			
SE	2.495	0.715	0.121	−1.15	0.405**	1		
OE	3.369	0.86	−0.391	−0.463	0.364**	0.403**	1	
PA	2788.47	1951.69	0.495	−0.7	0.333**	0.439**	0.376**	1

*n* = 937; M, mean; S.D., standard deviation. *p < 0.05, **p < 0.01; NE, neighborhood environment; PA, physical activity; SE, self-efficacy; OE, outcome expectations.

The results revealed significant positive correlations among neighborhood environment, self-efficacy, outcome expectations, and physical activity among older adults. Additionally, correlation analysis revealed significant associations between neighborhood environment and physical activity among older adults (*r* = 0.333, *p* < 0.01), neighborhood environment and self-efficacy (*r* = 0.405, *p* < 0.01), neighborhood environment and outcome expectations (*r* = 0.364, *p* < 0.01), self-efficacy and physical activity among older adults (*r* = 0.439, *p* < 0.01), outcome expectations and physical activity among older adults (*r* = 0.376, *p* < 0.01), and self-efficacy and outcome expectations (*r* = 0.403, *p* < 0.01). In summary, significant correlations were observed among all variables, providing preliminary evidence for the hypotheses proposed in this study.

### 3.4 Analysis of mediation effects

To verify the chain mediation effects of self-efficacy and outcome expectations in the relationship between neighborhood environment and physical activity among older adults, the AMOS 24.0 software package was used to conduct fit analysis of the conceptual chain mediation model. Following the mediation effect testing procedure proposed by Marsh et al. (2014). Table 4 presents the standard results of fit indices: χ^2^/df < 3, RMSEA < 0.08, CFI > 0.9, GFI > 0.9, NFI > 0.9, TLI > 0.9. The model parameters satisfied the fit requirements, indicating that the mediation model conceptualization between neighborhood environment and physical activity among older adults was reasonable.

**Table 4:** Questionnaire model fitting indicators.

Common indicators	χ2/*df*	RMSEA	CFI	GFI	NFI	TLI	IFI
Model	1.505	0.023	0.986	0.957	0.959	0.984	0.986

RMSEA, root mean square error of approximation; CFI, comparative fit index; GFI, goodness-of-fit index; NFI, normed fit index; TLI, Tucker-Lewis index. IFI, increased fit index.

**Figure 2:**
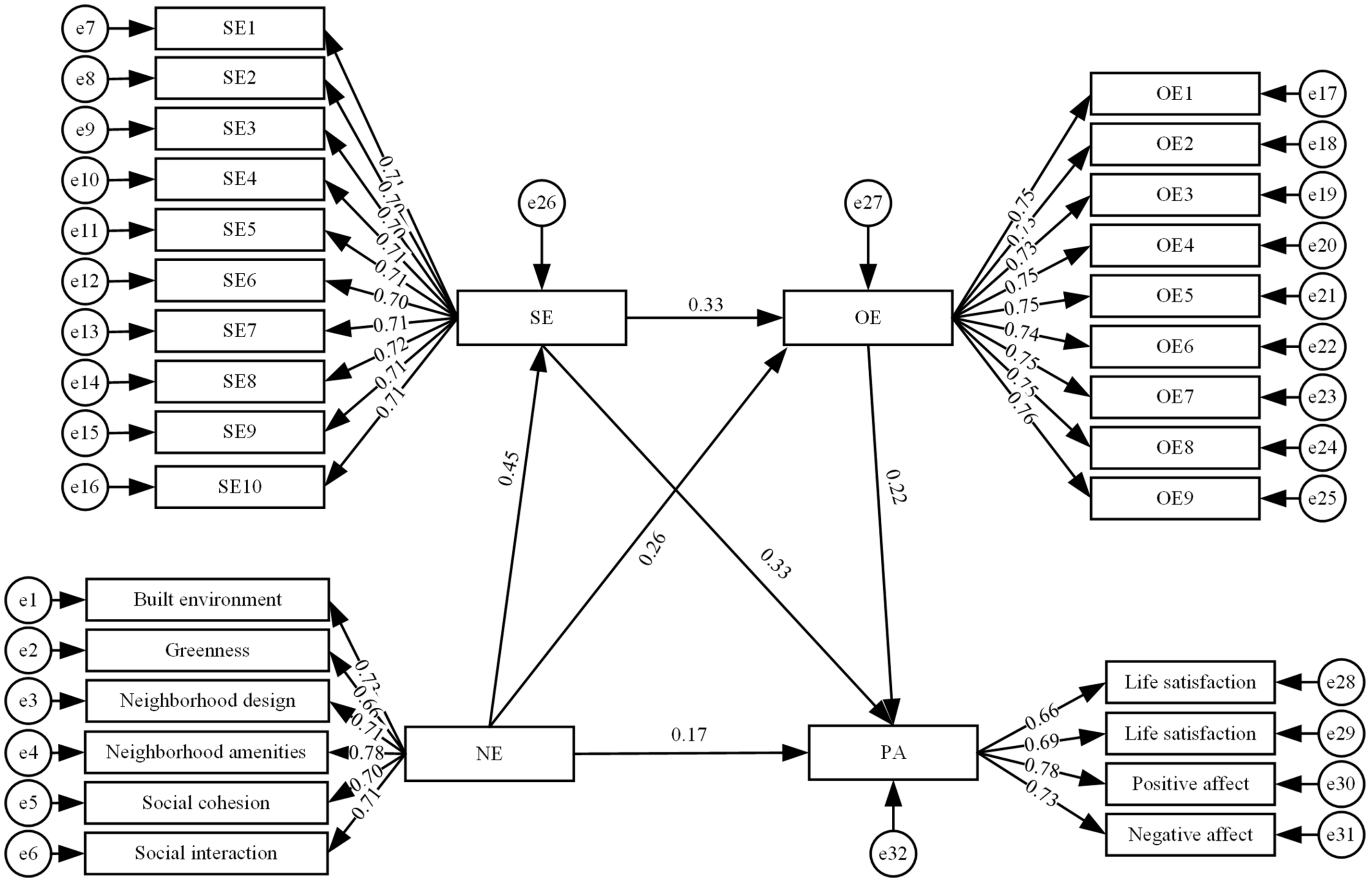
Intermediary model diagram.

As shown in Figure 2, the standardized path coefficient from neighborhood environment → physical activity among older adults was significant (*β* = 0.17, *p* < 0.05), indicating that neighborhood environment had a significant positive effect on physical activity among older adults, thus supporting hypothesis I. The path coefficients of neighborhood environment → self-efficacy (*β* = 0.45, *p* < 0.05) → physical activity among older adults (*β* = 0.33, *p* < 0.05) were significant, indicating that self-efficacy played a mediating role between neighborhood environment and physical activity among older adults, thus supporting hypothesis II. The path coefficients of neighborhood environment → outcome expectations (*β* = 0.26, *p* < 0.05) → physical activity among older adults (*β* = 0.22, *p* < 0.05) were significant, indicating that outcome expectations played a mediating role in the relationship between neighborhood environment and physical activity among older adults, thus supporting hypothesis III. The path coefficients of neighborhood environment → self-efficacy (*β* = 0.45, *p* < 0.05) → outcome expectations (*β* = 0.33, *p* < 0.05) → physical activity among older adults (*β* = 0.22, *p* < 0.05) were significant, indicating that self-efficacy and outcome expectations had a chain mediation effect between neighborhood environment and physical activity among older adults, thus supporting hypothesis IV.

A bias-corrected non-parametric percentile Bootstrap method was employed to assess the significance of individual mediation effects and confirm the mediating roles of self-efficacy and outcome expectations. Hayes (2009) recommended that the number of resampled datasets from the original sample in Bootstrap mediation effect testing should be at least 1,000. Bootstrap mediation effect test results indicate that if the Bootstrap test confidence interval (CI) does not contain zero, the indirect effect is established by Zhao et al. (2010). This study conducted mediation effect testing through 5,000 Bootstrap samples to determine the 95% confidence interval (CI). As shown in Table 5, the direct effect of neighborhood environment on physical activity among older adults was significant (direct effect = 0.166, 95% CI [0.067, 0.264]). The indirect effects included three significant mediation pathways: neighborhood environment → self-efficacy → physical activity (indirect effect = 0.146, 95% CI [0.103, 0.2]); neighborhood environment → outcome expectations → physical activity (indirect effect = 0.057, 95% CI [0.03, 0.095]); neighborhood environment → self-efficacy → outcome expectations → physical activity (indirect effect = 0.033, 95% CI [0.019, 0.051]). In summary, the Bootstrap 95% confidence intervals for all three mediation pathways did not contain zero, indicating that the path coefficients in this model were significant. These findings demonstrated that self-efficacy and outcome expectations served as mediators between neighborhood environment and physical activity among older adults, with self-efficacy and outcome expectations functioning as chain mediators.

**Table 5:** Test results of mediation effects.

Effect	Parameter	Estimate	BootSE	Bootstrap LLCI	Bootstrap ULCI
Direct effect	NE → PA	0.166	0.049	0.067	0.264
Indirect effect	NE → SE → PA	0.146	0.025	0.103	0.2
	NE → OE → PA	0.057	0.016	0.03	0.095
	NE → SE → OE → PA	0.033	0.008	0.019	0.051
Total effect	NE → SE → OE → PA	0.402	0.043	0.316	0.487

The Bootstrap sample size is set at 5000.

## 4 Discussion

This study confirmed that neighborhood environment exerts a significant positive predictive effect on physical activity among older adults, which is consistent with previous research findings (Bonaccorsi et al., 2020; Fisher et al., 2004). The results indicated that when communities provide better accessibility to sports facilities, improved pedestrian infrastructure, enhanced environmental safety, and positive social atmosphere, physical activity levels among older adults significantly increase. According to spatial perception theory, external environmental factors influence individual behavior, and the information and images individuals obtain from behavioral environmental factors can serve as the basis for behavioral decision-making and affect individual behavior (Song et al., 2024). Existing research indicates that neighborhood environment serves as an important venue for older adults to engage in physical activity and provides the foundational conditions for promoting leisure-time physical activity participation among older adults (Liang et al., 2025). As the ecological model emphasizes the significant influence of physical and social environments on individual behavior, neighborhood environment influences older adults’ behavior through multiple mechanisms. First is the affordance mechanism, whereby behavioral opportunities provided by the environment directly affect individual behavioral choices. Accessible sports facilities reduce the time and transportation costs for older adults to participate in physical activity, while well-developed pedestrian infrastructure provides safe and convenient conditions for daily physical activity (Oyeyemi et al., 2019). This contributes to enhancing the accessibility of outdoor physical activity and community-based sports activities for older adults, with such instrumental support elements serving as external facilitators for individuals to maintain healthy activities. In China, this affordance mechanism is particularly manifested in the allocation of public open spaces such as community plazas and park green spaces. The types of physical activity preferred by Chinese older adults, such as square dancing, Tai Chi, and health Qigong, mostly possess collective and social characteristics, requiring relatively spacious activity venues and appropriate audio equipment support. Therefore, whether communities provide sufficient plaza space and whether they are equipped with fitness equipment and activity facilities directly affects the implementation of physical activity. Second is the cue mechanism, whereby favorable exercise environments themselves constitute visual and interpersonal atmospheres that promote physical activity (Yang et al., 2022), reminding and encouraging older adults to engage in exercise, stimulating their exercise intentions, and making them more inclined to engage in outdoor activities during leisure time, thereby avoiding sedentary behavior and mobile phone addiction. In Chinese neighborhoods, this cueing mechanism is often reinforced through role modeling and group effects. When older adults observe their neighbors and peers actively participating in various physical activities within the community, they are more likely to generate the psychological cue that they should also get moving. Chinese older adults place high value on peer relationships and collective belonging; the active exercise atmosphere within the community serves not only as a visual cue but also as an embodiment of social norms and cultural identity. Third is the support mechanism, whereby safe and appropriate road layouts and transportation facilities help reduce the difficulty and risks associated with older adults’ participation in sports activities (Chang, 2020), enhance aesthetic qualities and positive experiences, stimulate enthusiasm for sports participation and autonomous agency, and enable older adults to actively and voluntarily participate in physical activities in the community during leisure time. In Chinese neighborhoods, this support mechanism is manifested not only at the material environment level but also in organized and institutionalized service provision. Many neighborhoods regularly organize fitness activities, sports competitions, and health lectures through neighborhood committees or homeowners’ associations, providing older adults with structured participation opportunities. Neighborhood environment not only provides objective conditions for physical activity but, more importantly, creates an opportunity structure that promotes an active lifestyle, making healthy behavior a natural choice rather than a deliberate effort for older adults.

Neighborhood environment not only directly influences physical activity among older adults but also indirectly affects their physical activity by positively impacting self-efficacy, which is consistent with previous research findings (Wei et al., 2025). According to Bandura’s social cognitive theory, self-efficacy serves as the core driving force for behavior change, influencing not only whether individuals choose to engage in a particular behavior but also determining their level of persistence and effort when facing difficulties. Neighborhood environment enhances exercise self-efficacy among older adults through multiple pathways. First is mastery experience, whereby accessible facilities and safe environments reduce the objective difficulty of exercise, enabling older adults to more easily acquire successful exercise experiences, thereby strengthening their confidence in their own exercise capabilities. Research demonstrates that successful exercise experiences constitute the most powerful pathway for enhancing self-efficacy, as they not only provide direct evidence of individual capability but also promote dopamine release, generating positive emotional experiences. This sense of pleasure further reinforces individuals’ positive cognition of exercise and participation motivation (Allison and Keller, 2004). Second is vicarious experience, whereby favorable community exercise atmospheres provide increased opportunities for vicarious learning. Older adults indirectly enhance their assessment of their own exercise capabilities by observing others’ successful exercise experiences, particularly the successful experiences of those with similar conditions to themselves. Third is verbal persuasion, whereby positive social support and encouragement provide older adults with positive feedback from significant others, further strengthening exercise self-efficacy. Fourth is emotional arousal, whereby favorable neighborhood environments can reduce exercise-related anxiety and fear (Beenackers et al., 2011), create positive emotional states, and thereby indirectly enhance self-efficacy. Neighborhood environment creates favorable conditions for the development of self-efficacy among older adults by providing diverse exercise opportunities and supportive social contexts. Particularly for older adults with limited exercise experience, favorable neighborhood environments can provide low-risk, high-support exercise experiences, helping them gradually establish exercise confidence.

Concurrently, neighborhood environment can also indirectly influence physical activity among older adults by positively affecting outcome expectations. According to social cognitive theory, individual behavior is not only directly influenced by environmental factors but, more importantly, behavior change is achieved through the mediating role of cognitive processes. When neighborhood environments provide better support for physical activity (such as well-equipped sports facilities, safe pedestrian environments, and accessible fitness venues), older adults are more likely to develop positive outcome expectations, which is consistent with previous research findings (Peters et al., 2020). Based on positive expectations of exercise outcomes generated by neighborhood environment quality, these outcome expectations encompass multiple dimensions: The health expectation dimension manifests as older adults expecting to achieve physical health improvements through physical activity, such as weight control, cardiovascular function enhancement, and muscle strength augmentation (Breda and Watts, 2017). Favorable neighborhood exercise environments make such health expectations more concrete and achievable. The social expectation dimension is characterized by well-equipped community sports facilities providing social interaction platforms for older adults, enabling them to expect expanded social networks, enhanced neighborhood relationships, and increased social support through physical activity (Gao et al., 2015). The psychological expectation dimension refers to safe and aesthetically pleasing exercise environments leading older adults to expect that physical activity can bring psychological benefits such as emotional regulation, stress relief, and self-actualization (Wood et al., 2017). When individuals believe that a particular behavior can yield valuable outcomes, stronger behavioral intentions and motivation are generated. Existing research has confirmed that outcome expectations constitute an important predictor of physical activity among older adults. Older adults who hold positive outcome expectations regarding physical activity are more likely to actively seek activity opportunities, overcome participation barriers, and maintain regular exercise habits. These multidimensional positive outcome expectations function synergistically to form the intrinsic motivation driving physical activity behavior, thereby promoting the occurrence of actual physical activity behavior (Resnick et al., 2000).

A relatively stable systematic association exists between self-efficacy and outcome expectations, which is consistent with previous research findings (Anderson et al., 2006). The influence of neighborhood environment on physical activity among older adults involves intrinsic psychological mechanisms, whereby favorable neighborhood environments first enhance self-efficacy among older adults, subsequently strengthen their positive expectations regarding physical activity outcomes, and ultimately promote the occurrence of physical activity behavior. This finding reveals the intrinsic mechanisms through which neighborhood environmental factors are transformed into behavior change via cognitive appraisal processes. Self-efficacy, as an individual’s belief in their capability to accomplish specific behaviors, plays a critical role in physical activity among older adults. When communities possess accessible fitness facilities, safe and comfortable pedestrian environments, and adequate open spaces, older adults are more likely to acquire successful activity experiences, and such positive experiences can enhance their confidence in their own activity capabilities (Carlson et al., 2012). Favorable neighborhood environments reduce objective barriers to activity participation, diminish older adults’ concerns about risks such as falls and getting lost, and enable them to believe they possess the capability to safely engage in various types of physical activity. Existing research has demonstrated that environmental accessibility and safety constitute important factors influencing self-efficacy among older adults (Kostka and Jachimowicz, 2010). This study further confirms that neighborhood environment can effectively enhance activity confidence among older adults by providing convenient conditions and reducing activity risks. Outcome expectations represent individuals’ anticipated judgments regarding the potential consequences of specific behaviors, reflecting older adults’ cognition of the value of physical activity. Research has found that enhanced self-efficacy can facilitate older adults in developing more positive outcome expectations. When older adults possess confidence in their activity capabilities, they are more inclined to believe that physical activity can yield positive outcomes such as health improvements, enhanced social connections, and elevated quality of life. This cognitive transformation aligns with Bandura’s social cognitive theory, which posits that individuals’ capability beliefs influence their expectations regarding behavioral outcomes (Bandura, 2001). Specifically, older adults with higher self-efficacy are more likely to focus on the positive effects of physical activity while overlooking or minimizing expectations of difficulties and risks, thereby forming the dual cognition of “I can do it and doing it will bring benefits.” These positive outcome expectations further strengthen motivation for physical activity, making older adults more willing to actively participate in various activities. A progressive relationship exists between self-efficacy and outcome expectations, with both collectively constituting the psychological transformation pathway from environment to behavior. Neighborhood environment, as an external condition, provides the physical foundation for older adults’ activities; self-efficacy, as a capability assessment, enables older adults to believe they can utilize these conditions to engage in activities; outcome expectations, as a value judgment, enable older adults to recognize the significance and benefits of activities. These three elements operate sequentially, forming a complete cognitive chain of “environment provides possibilities, individuals assess feasibility, and individuals recognize necessity.” This process demonstrates that environmental factors do not simply and directly determine behavior but are gradually transformed into behavioral motivation by influencing individuals’ capability beliefs and outcome expectations. Compared with previous studies, this research not only validates the independent effects of environmental and cognitive factors on physical activity but, more importantly, reveals the sequential relationships among multiple cognitive factors and their synergistic effects in behavior change, providing new perspectives for deeply understanding the influencing mechanisms of physical activity among older adults and offering theoretical foundations for conducting targeted interventions in the future.

## 5 Implications and limitations

### 5.1 Implications

This study explores the mechanisms through which neighborhood environment influences physical activity among older adults by introducing self-efficacy and outcome expectations as mediating variables, thereby extending existing research on the relationship between neighborhood environment and health behaviors among older adults. Neighborhood environment plays a crucial role in enhancing self-efficacy and strengthening outcome expectations. Through high-quality neighborhood environmental support, older adults gain successful experiences and vicarious learning opportunities during physical activity participation, which improves their confidence in and judgment of their own exercise capabilities. Simultaneously, through enriching activity experiences and positive exercise atmospheres, they perceive the physical and psychological benefits derived from physical activity, establishing expectations for positive outcomes such as health promotion, social interaction, and emotional regulation. Ultimately, this accumulation and mutual reinforcement of cognitive resources collectively enhance physical activity levels among older adults. By examining the chain mediating role of self-efficacy and outcome expectations, this study provides new perspectives and practical approaches for promoting physical activity among older adults. First, from a health promotion intervention perspective, traditional physical activity interventions for older adults have predominantly focused on single approaches such as environmental modifications or health education. However, this study confirms the effectiveness of an integrated strategy combining environmental optimization with cognitive intervention. When promoting physical activity levels among older adults, more comprehensive intervention measures are needed that synergistically consider multiple factors including neighborhood environment construction, self-efficacy cultivation, and outcome expectation reinforcement. Community health and sports management departments should establish systematic cognitive intervention mechanisms that help older adults accumulate successful experiences and build exercise confidence through practical participation via stratified and graded exercise guidance, peer demonstration and sharing, skill training and coaching, and positive feedback assessment. Simultaneously, through health education, collective activity organization, and visualization of benefits, outcome expectations should be strengthened to maximize the promotional effects of neighborhood environment on physical activity. Second, from the perspective of practical significance for community development, understanding the chain mediating role of self-efficacy and outcome expectations contributes to optimizing the planning, design, and service provision of age-friendly communities. Urban planners and community managers can consciously integrate cognitive support elements into environmental transformation processes, fully consider the convenience of use for older adults when allocating fitness facilities, and emphasize the creation of social interaction spaces in activity venue design, enabling the neighborhood environment to not only provide material support but also serve as a vehicle for stimulating exercise confidence and strengthening benefit expectations among older adults. Communities should regularly organize diverse collective fitness activities to cultivate a strong exercise culture atmosphere, allowing older adults to gain a sense of collective belonging and achievement satisfaction through participation, and enhance their own self-efficacy and outcome expectations by observing health improvements in their peers. Finally, from a policy-making perspective, attention should be paid to the dynamic development of cognitive states among older adults, establishing an integrated intervention system with multi-departmental coordination. Differentiated intervention strategies should be implemented for older adults with varying cognitive levels. Regular health lectures and exercise skill training sessions with enriched content can be conducted, and professional community health instructors can be deployed to provide continuous support, thereby promoting more comprehensive physical and mental health development among older adults and encouraging them to maintain stronger exercise motivation and higher activity levels in daily life. This aims to achieve the goal of healthy aging and provide scientific evidence and practical guidance for advancing the development of the national fitness initiative.

### 5.2 Limitations

Although this study has yielded valuable findings, certain limitations remain that require further refinement and deepening in future research. First, this study employed a cross-sectional survey design. While advanced statistical methods were utilized to infer the chain mediation effects of self-efficacy and outcome expectations, the temporal sequence and true causal relationships among neighborhood environment, self-efficacy, outcome expectations, and physical activity could not be definitively established. Future research should adopt longitudinal tracking designs to more accurately verify the dynamic change processes and causal relationships of chain mediation mechanisms through multi-timepoint data collection. For example, by observing changes in cognitive states and physical activity levels among older adults before and after neighborhood environment improvements, or by comparing the effects of different intervention measures through randomized controlled trials, more robust evidence can be provided for causal inference. Second, this study primarily relied on self-report questionnaires for data collection, which may introduce issues such as social desirability bias and common method bias. Future research should employ diversified data collection methods, such as behavioral observation, objective environmental measurement, and physiological indicator monitoring, to enhance the objectivity and reliability of research findings. Particularly for the physical activity variable, consideration should be given to using exercise monitoring devices such as accelerometers and pedometers to record objective activity data rather than relying solely on subjective reports. Third, although the sample in this study possesses certain representativeness, it remains primarily concentrated in 12 communities in Chengdu, and the generalizability of the sample requires further verification. Future research should expand the sample scope to include different regions, different development levels, and different types of communities to enhance the external validity and practical application value of research findings. Finally, this study primarily focused on two mediating variables, self-efficacy and outcome expectations, but the mechanisms through which neighborhood environment influences physical activity among older adults may be more complex. Future research could consider incorporating additional potential mediating variables, such as social support, behavioral intention, environmental perception, and health motivation, to construct more comprehensive mediation models. Simultaneously, possible moderating variables such as age, gender, health status, and exercise history could be explored to more precisely understand the differentiated mechanisms through which more precisely understand the differentiated mechanisms neighborhood environment influences physical activity across different populations.

## 6 Conclusion

This study revealed the influence of neighborhood environment on physical activity among older adults and conducted an in-depth analysis through the chain mediation effects of self-efficacy and outcome expectations. The results demonstrated that neighborhood environment exerts a positive influence on physical activity among older adults. Older adults residing in communities with better environmental support not only exhibited higher levels of physical activity but also achieved significant improvements in self-efficacy and outcome expectations. The study found that self-efficacy played a significant mediating role between neighborhood environment and physical activity. Neighborhood environment, through providing well-equipped sports facilities, safe activity venues, and accessible fitness services across multiple dimensions, helps older adults develop more positive and stable exercise self-efficacy. In favorable neighborhood environments, older adults continuously reinforce their confidence in and assessment of their own exercise capabilities through convenient facility use, successful exercise experiences, and sustained activity participation, thereby enhancing exercise self-efficacy and behavioral control, which provides a solid psychological foundation for participating in and maintaining physical activity. Outcome expectations similarly played a significant mediating role between neighborhood environment and physical activity. Neighborhood environment provides older adults with abundant opportunities for physical activity participation and positive exercise experiences. Through observing health improvements in others, experiencing favorable exercise atmospheres, and receiving community support and encouragement, individuals’ expectation levels regarding positive outcomes of physical activity are significantly elevated. Older adults with stronger outcome expectations can more clearly recognize the multiple benefits of physical activity in health promotion, social interaction, and emotional regulation, maintain positive participation motivation when facing exercise barriers, more easily obtain anticipated benefits and satisfaction from physical activity, and thereby promote the sustained implementation of physical activity behavior. This study verified for the first time the chain mediation effects of self-efficacy and outcome expectations between neighborhood environment and physical activity among older adults. Neighborhood environment not only directly improved physical activity levels among older adults but also further enhanced individuals’ confidence in exercise capabilities and cognition of activity benefits through promoting the enhancement of self-efficacy and the formation of outcome expectations. By effectively enhancing exercise self-efficacy and strengthening positive outcome expectations, older adults can better utilize community resources, maintain firm participation beliefs when facing exercise challenges, and thereby achieve comprehensive improvements in physical activity levels. The chain mediation model proposed in this study integrates the relationships among self-efficacy, outcome expectations, and physical activity among older adults, providing new perspectives for more comprehensively understanding how neighborhood environment influences physical activity among older adults through cognitive mediation mechanisms. This finding not only enriches existing literature but also provides theoretical foundations for the application of neighborhood environment construction in the fields of health promotion and behavior change among older adults. The research findings hold significant practical guidance implications for community health management policy formulation, urban planning and design optimization, and promotion of national fitness program development.

## Data Availability

The datasets presented in this article are not readily available because the cross-sectional design limits causal inference, self-reported measures may introduce bias, and the sample from specific regions may limit generalizability to diverse older adult populations. Requests to access the datasets should be directed to Qingqing Yang, yqq177123456@163.com.
